# Atopic Dermatitis in Animals and People: An Update and Comparative Review

**DOI:** 10.3390/vetsci4030037

**Published:** 2017-07-26

**Authors:** Rosanna Marsella, Anna De Benedetto

**Affiliations:** 1 Department of Dermatology, College of Medicine, University of Florida, 4037 NW 86 Terrace, Gainesville, FL 32606, USA; adebenedetto@dermatology.med.ufl.edu; 2 Department of Small Animal Clinical Sciences, College of Veterinary Medicine, University of Florida, 2015 SW 16th Avenue, Gainesville, FL 32610, USA

**Keywords:** atopic dermatitis, skin barrier, atopic march, dogs, cats, horses, humans

## Abstract

Atopic dermatitis is an extremely common, pruritic, and frustrating disease to treat in both people and animals. Atopic dermatitis is multifactorial and results from complex interactions between genetic and environmental factors. Much progress has been done in recent years in terms of understanding the complex pathogenesis of this clinical syndrome and the identification of new treatments. As we learn more about it, we appreciate the striking similarities that exist in the clinical manifestations of this disease across species. Both in animals and people, atopic disease is becoming increasingly common and important similarities exist in terms of immunologic aberrations and the propensity for allergic sensitization. The purpose of this review is to highlight the most recent views on atopic dermatitis in both domestic species and in people emphasizing the similarities and the differences. A comparative approach can be beneficial in understanding the natural course of this disease and the variable response to existing therapies.

## 1. Introduction

Increasing awareness exists with respect to the similarities between animal and human diseases and the appreciation of how a comparative approach is useful to improve our understanding of pathogenesis and the development of new treatments [1,2]. Presently, it appears that allergic diseases in both animals and people are diagnosed with increased frequency, possibly as the result of changes in the environment, the exposure to microbes and parasites, and other lifestyle changes. Manifestations of atopic disease (e.g., atopic dermatitis, rhinitis, asthma) in people are common, particularly in westernized countries [3]. Atopic disease is linked to an increased predisposition to the development of allergies. In humans, the progression from cutaneous manifestations of atopic disease to respiratory signs is known as “atopic march” [4].

Dogs, for the most part, tend to remain in the cutaneous stage of the atopic disease. Similarly to humans, atopic dermatitis (AD) in dogs is becoming increasingly common [5]. Much progress has been done in the past decade in terms of understanding the complex pathogenesis of this condition in dogs. As our knowledge increases, the similarities and differences of the canine disease compared to the human disease are becoming more evident. Manifestations of atopic disease (both the cutaneous and the respiratory component) exist in other domestic animals, like cats and dogs, although much less is known in these species.

The purpose of this comparative review article is describe the most recent information available on AD in dogs, cats, horses, and people, both in terms of pathomechanisms and new therapies. Some of these aspects may be the key to better understand this complex clinical syndrome and possibly help with the identification of new approaches.

## 2. Atopic Dermatitis in Dogs: A Clinical Syndrome by Many Triggers

Much progress has been done in the past decades on our understanding of this complex syndrome. Currently, the term canine AD is used to define a clinical disease that is associated with characteristic clinical signs and the presence of allergen specific IgE [6]. Based on the clinical experience of a variable clinical response when targeted treatments are used, it is increasingly clear that different pathways may achieve clinical signs of AD. Thus, AD is not to be viewed as a single entity, but more as the descriptive term for a clinical syndrome.

Currently, in veterinary medicine, the term AD is frequently used as synonymous as environmental allergic skin disease and the presence of allergen-specific IgE are considered one of the criteria for clinical diagnosis of AD. The term atopic-like dermatitis (or intrinsic AD) is reserved for dogs that have clinical signs of AD, but no detectable allergen-specific IgE. It is unclear whether this presentation represents the early stage of AD or a different subtype of this syndrome. Clinically, the classic atopic dogs and the atopic-like dog are indistinguishable, creating a potential additional challenge for the clinician in terms of therapy. Currently, we do not know if this subtype of dogs are less responsive to drugs used for the management of pruritus, but we do know that they are not amenable to allergen-specific immunotherapy since we cannot demonstrate an allergenic trigger for their disease.

In the past, in veterinary medicine, the term AD was used when the allergic trigger was environmental allergens. Currently, we have increased awareness that food driven skin disease may look indistinguishable from the one triggered by pollen or other environmental allergens. Thus, the traditional separation between “food allergy” and AD is no longer an appropriate one. AD is a clinical diagnosis and does not refer to the nature of the allergenic trigger. It is also important to point out that food-induced dermatitis may manifest in a multitude of ways and that AD is only one of the possible manifestations. Others may include, but are not limited to, urticarial plaques or vasculitis.

## 3. Canine AD and Skin Barrier Abnormalities

The topic of the role of skin barrier abnormalities in canine AD is relatively new compared to what has been known in human medicine for decades. Yet, there is increasing evidence that some skin barrier dysfunction exists, although it is still unknown whether a primary skin barrier defect exists [7]. All of the studies so far have focused on comparing atopic dogs with healthy controls and have not compared the changes observed in atopic skin to what may be present in dogs with other skin diseases. This is of great importance before any definitive statement can be made about the specificity of the changes as it pertains to AD. It is indeed known that inflammation can worsen the skin barrier, thus, it is important to remember that some of the changes observed in inflamed atopic skin may be, at least in part, due to the presence of inflammation, whether that is clinically evident or only histologically present.

Currently, several studies have reported decreases in ceramides [8], alterations in filaggrin expression [9], and higher expression of the enzymes involved in filaggrin metabolism (such as calpain-1, caspase 14, and matriptase) [10,11] and increased Transepidermal Water Loss (TEWL) in dogs with AD [12]. The changes in ceramide content have been linked to the increased TEWL and impaired skin barrier [13]. The amount of total lipids and ceramides including all ceramide classes are significantly lower in both lesional and non-lesional skin of atopic dogs compared to normal skin, with the reduction being more severe in lesional skin [14]. Interestingly, no connection between lipid composition and predilection sites for canine atopic dermatitis lesions [12]. The awareness of decreased epidermal lipids has triggered the focus on topical application of lipid emulsions as a strategy for skin barrier repair and improvement of clinical signs [15]. Several studies have reported on the beneficial effect of topical application of ceramide-based formulations on both the restoration of lipid composition in the stratum corneum [16,17], as well as improvement of clinical signs in dogs with AD [18].

## 4. Interplay between Skin Barrier Dysfunction and Allergic Inflammation

The relationship between skin barrier dysfunction and allergic sensitization and inflammation is a two way street: skin barrier function is worsened by inflammation [19] and the worse is the skin barrier, the more propensity exists toward allergic sensitization. The negative effect of T helper 2 (Th2) cytokines on skin barrier integrity is well known [20]. The fact that the skin barrier is impaired determines how allergens are processed and promotes a Th2 response. Increased propensity for allergic sensitization has been demonstrated in an experimental model of canine AD after removal of the stratum corneum by tape stripping [21]. In a controlled study it was demonstrated that dogs in which the stratum corneum was removed became sensitized faster and with higher IgE levels than dogs in which the stratum corneum was left in place highlighting the importance of impairment of skin barrier as one of the factors that facilitates epicutaneous sensitization to allergens [19].

Exposure to the antigen after the removal of the stratum corneum causes Langerhans cells to migrate and the antigen presentation gives, predominantly, a Th2 response [22]. Thus, the skin barrier defect is linked to the propensity toward allergic sensitization after epicutaneous exposure in atopic patients. Currently we know that increased Thymic Stromal Lymphopoietin (TSLP) expression occurs in the skin of atopic dogs, although no study has documented that this is the mechanism underlying epicutaneous sensitization in dogs after skin damage [23]. TSLP certainly has the ability to promote a Th2 response [24] and modulate the response of dendritic cells upon allergen exposure. Increased expression of TSLP may be linked to skin damage or stimulation by *Staphylococcus* via Toll-like receptors [25]. This is an important consideration as the relationship between microbes and AD is a complex one and a clinically relevant one.

## 5. Canine AD and Microbiome

The complex interaction between the skin barrier and host microorganism interaction is an area of growing interest in veterinary dermatology [7]. This is an emerging area of research in veterinary dermatology [26] as only a few studies have been published so far. The microbiome refers to the combination of microorganisms and their genetic material.

The vast majority of these organisms are harmless to the host and many of them are actually beneficial. The microbiome is, therefore, of crucial importance to the host. For one, the “beneficial” organisms occupy a niche, avoiding the invasion by more pathogenic bacteria. Furthermore, these microorganisms interact with the innate and adaptive immune system and educate the immune system [27]. The microbiome is dynamic and changes over the course of the life of an individual and is affected by both endogenous and exogenous factors. Biodiversity (variability among living organisms from all sources) is essential to educate the immune system and loss of biodiversity has been linked to the increased development of chronic inflammatory and allergic diseases in recent years. This is because the microbiome has an important function in educating the immune system and diversity is needed to achieve tolerance [28]. Most of the interaction between the commensal microbiota with the innate immune system aims to prevent overgrowth and infection by obtaining a delicate balance between regulation and homeostasis. Allergies are driven by an abnormal response to otherwise harmless antigenic triggers, leading to chronic inflammation. The “hygiene hypothesis” had suggested that a lack, or a decrease, of microbial exposure leads to Th2 dysregulation and allergy development. This theory has now been revised to something related, called the “microflora hypothesis” that suggests that alterations in the microbiota disrupt immune tolerance. Therefore, loss of biodiversity due to lifestyle changes, whether dietary or environmental, promotes the development of an atopic state, which then promotes a chronic inflammatory response [29].

In one published study evaluating the differences between healthy and allergic dogs [30], it was reported that allergic dogs have less biodiversity than normal dogs with a larger number of coagulase-positive *Staphylococcus* compared to healthy dogs. It is currently unclear whether this lower diversity is a cause or an effect. The study done by Rodrigues et al. in atopic dogs did not look at disease severity or evolution of atopic dermatitis lesions. Instead, it was just a snapshot in time to evaluate basic differences between healthy and allergic skin. This is an important starting point to see if there are fundamental differences between healthy and atopic canine skin.

Veterinarians are constantly challenged with the development of secondary bacterial infections in dogs with AD [31]. In the past, the role of *Staphylococcus* was always considered secondary. Currently, there is debate whether *Staphylococcus* can actually be a “cause” of AD and not simply a complicating factor that leads to exacerbation of clinical signs of AD [32]. Dysbiosis is observed during flares of canine AD and is partly normalized during treatment of AD and remission of clinical signs [33]. Treatment restored bacterial diversity with decreased proportions of *Staphylococcus* species, concurrent with decreased canine atopic dermatitis severity. Skin barrier function, as measured by corneometry, pH, and TEWL also normalized with treatment. Another study reported on skin microbiome changes in an experimental model of canine AD during allergen-induced flares [34]. Such a study showed that allergen challenge in sensitized dogs led to bacterial dysbiosis with increased abundance of *S. pseudintermedius* at the site of lesion induction.

## 6. Clinical Signs of Canine AD

**Figure 1:**
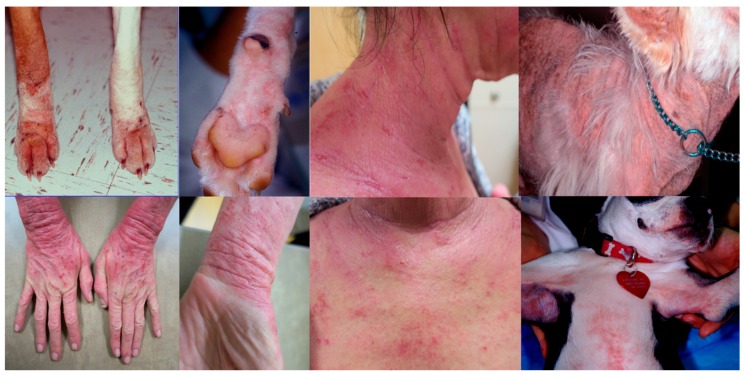
Extremities, neck, and chest are frequently affected by erythema and pruritus in both dogs and people.

**Figure 2:**
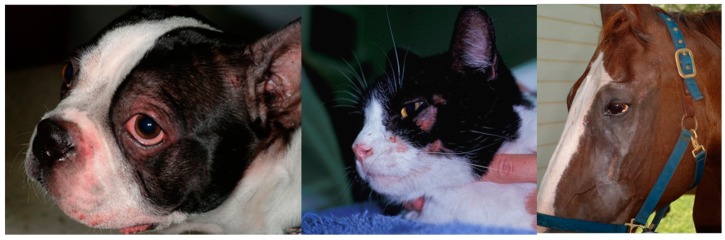
The face is a common area of pruritus across species. Erythema and pruritus are main signs and frequently lead to excoriations.

Canine AD is characterized by erythema and pruritus that preferentially affects some body areas [35]. Those areas include the muzzle, neck, chest, periocular areas, the pinnae, the antebrachial area, and the axillary and inguinal areas (Figure 1 and Figure 2). Interestingly, these areas have been found to have increased permeability compared to other body areas [12] so it could be speculated that this may be a reason for why they tend to be preferentially affected. Pruritus is a consistent characteristic of this condition and it tends to worsen once secondary infections develop. Canine AD is aggravated by other allergies, such as flea allergy, and many patients may have overlapping signs of AD and flea allergy. The presence of multiple triggers plays a very important role in the management of pruritus as all triggers are additive.

AD develops in young individuals and it is initially, in most cases, seasonal. As time progresses, AD has the propensity to become year-round and to progressively increase in severity. Thus, the course is chronically progressive in the majority of patients. Secondary infections, both bacterial and yeast, frequently develop as self-trauma and inflammation makes the skin more vulnerable.

## 7. Diagnosis of Canine AD

Despite many efforts to identify a “diagnostic test” for canine AD, the diagnosis remains clinical. A variety of clinical criteria [36,37] have been considered over the years with variable sensitivity and specificity. Regardless of the criteria, the diagnosis is based on history, clinical signs, and exclusion of other pruritic diseases. Detection of allergen-specific IgE is considered a minor criterion. Thus, allergy testing to detect allergen-specific IgE, both serology and intradermal skin tests, cannot be used for diagnostic purposes as they do not have a great ability to discriminate between normal and atopic patients [38]. Allergy testing is best used to identify allergens to include in allergen-specific immunotherapy once a clinical diagnosis of AD has been made.

## 8. Clinical Management of Canine AD

Due to the multifactorial nature of this disease, management frequently requires a multimodal approach to decrease pruritus below the threshold of clinical signs. Concurrent allergies and secondary infections add to the pruritus and need to be controlled in order to minimize the pruritus and just focus on the portion due to the atopic disease.

### 8.1. Management of Acute Flares

Acute flares are best handled by (1) identifying the most immediate trigger factors (e.g., fleas, specific foods); and (2) using a treatment that can provide immediate relief decreasing inflammation and pruritus. The persistence of inflammation and the skin damage caused by self-trauma can have rapidly negative effects not only on the comfort level of the animals, but also to the development of the secondary infections, which can further complicate and increase the severity of the clinical signs. While glucocorticoids have been used for a long time and are well known treatments with their pros and cons, the use of a JAK inhibitor, like oclacitinib, is still relatively new. Oclacitinib has been demonstrated to be effective and rapid in providing relief to affected animals and it is considered a suitable alternative to the use of glucocorticoids to provide fast control of clinical signs [39]. The speed of action is considered comparable to that of oral glucocorticoids [40]. The recommended regimen is 0.4–0.6 mg/kg orally twice daily for the first two weeks, followed by once daily for subsequent therapies. Although oclacitinib may not work in all atopic patients, it is, overall, a very effective therapy and well-tolerated treatment. Many patients may show a worsening of clinical signs when switched from the twice-daily to the once-daily treatment, which typically levels out over time. In addition to the lack of adverse effects, like polyuria and polydipsia, oclacitinib has another advantage over glucocorticoids as it does not appear to have a negative impact on intradermal skin testing, thus, it could be used in the short-term to help make the patient comfortable while working up the triggers, such as environmental allergens. Unfortunately the benefits of relief provided by oclacitinib are typically short-lived once the medication is discontinued and clinical signs rapidly return, sometimes even at a higher level than before the initiation of therapy (rebound). It is important to note that oclacitinib is not approved for use in dogs younger than 12 months of age and, thus, it cannot be used in very young atopic patients.

For patients that cannot take either oclacitinib or other therapies, another option is a recently-released biologic. This is a subcutaneous injection of the caninized anti-cIL-31 monoclonal antibody and is aimed at blocking IL-31, which is a mediator of pruritus in dogs [41]. Whether IL-31 is a main cytokine in canine AD is still under investigation. One study had failed to detect IL-31 in the skin of atopic dogs altogether, and another study detected IL-31 in the serum of only 57% of atopic dogs. Thus, although IL-31 injections can induce pruritus in dogs, it is not clear yet that this is a critical cytokine in canine AD. IL-31 monoclonal antibody provides an approximately 60% reduction in pruritus according to owners and up to 50% decrease of CADESI in most atopic patients [42]. The benefit typically lasts one month, although great variability is seen in clinical settings. This therapy appears to be well-tolerated with minimal propensity for triggering immunity [42].

As part of the acute flare management, it is important to use topical therapy as an adjunctive strategy to decrease pruritus and sooth the skin. Topical glucocorticoids can also be used to provide fast relief, particularly in patients with localized disease. If infections are present, they need to be addressed to decrease pruritus and allow maximum benefit of anti-inflammatory treatments.

### 8.2. Medium to Long-Term Management

For the medium term control of the disease, many clinicians chose to use cyclosporine. The benefit of cyclosporine therapy is not evident for the first 3–4 weeks, thus, this type of approach requires the use of another faster-acting therapy while waiting for the benefit of cyclosporine. Like other immunomodulating therapies, cyclosporine may increase the risk for infections when used for prolonged periods of time. Despite this, cyclosporine is considered, overall, to be a safe treatment for medium- to long-term use. The most common adverse effect when prescribed at 5 mg/kg once daily is gastrointestinal, ranging from vomiting to diarrhea and decreased appetite. In some dogs a papillomatous dermatitis may develop, which is typically responsive to a decrease of the dose and antibiotic therapy.

Oclacitinib can also be used for long-term therapy as well, and it appears to be safe [43]. However, for patients requiring medication for many months/year it is always prudent to find alternative therapies when treatment is needed for extended periods of time. The long-term strategy is typically composed of anti-inflammatory treatments (e.g., cyclosporine, oclacitinib, or glucocorticoids) in combination with immunotherapy to modulate the hypersensitivity reaction and minimize future flare-ups.

Allergen specific immunotherapy (ASIT) is still considered the best long-term approach for young animals with symptoms present for many months/years [44]. Although allergen-specific immunotherapy is typically presented as “expensive”, when compared to the cumulative cost of other forms of management (e.g., cyclosporine in a large-breed dog) it is actually cost effective, as it can decrease the frequency of infections and, therefore, reduce the use of antibiotics and the risk of resistance, as well as the need for other medications. In human medicine, allergen-specific immunotherapy has been demonstrated to alter the course of the disease and decrease the number of sensitizations in the long run. Whether this is applicable to dogs is currently unknown. Despite the fact that allergen-specific immunotherapy has been used for many years, there are few published studies, particularly controlled ones. Insufficient information is currently available to indicate which protocol is the best one, although it appears that higher doses and allergen-specific regimes seem to provide better success versus low dose and pre-mixed regimes. While the traditional route of administration for allergen-specific immunotherapy has been the subcutaneous route, more recent studies have also showed safety and efficacy of sublingual immunotherapy [45]. This route should be considered in patients that have had adverse effects with the injections. The improvement with sublingual immunotherapy may be noticeable in the first six months and, therefore, may be considered faster than the traditional subcutaneous injections. Most patients require adjunctive therapy in the first few months of therapy; therefore, drugs like glucocorticoids, cyclosporine, or oclacitinib may be used while building up the allergen-specific immunotherapy. An indirect measurement of the effect is noticed by the decreased need for medications to maintain control of the signs.

An approach accepted in human medicine to reduce flare-ups is the proactive treatment of areas where the patient typically develops lesions, even when the skin appears to be clinically normal. This approach has been tested in a small, randomized, controlled study in atopic dogs with encouraging results. In this trial hydrocortisone aceponate spray was applied to areas prone to lesions two days/week and that led to a four times longer relapse time [46]. Monitoring of cutaneous atrophy should be considered in the long term.

Finally, the use of essential fatty acids, either orally or topically, should be integrated in the long-term management of atopic patients. This type of supplementation requires time to produce a beneficial effect, but it has been proven to increase and restore some of the lipid abnormalities in the epidermis. Topical application of sphingolipid emulsions can also lead to improvement and should be considered as part of adjunctive therapy rather than monotherapy. Similar consideration is for the use of antihistamines, which are best used before the beginning of the allergy season and more with the goal of adjunctive therapy to minimize the need for other medications, rather than a rescue drug once an acute flare up has developed.

In conclusion, the management of canine AD is multimodal and should be tailored to the individual patient considering the age, the duration of symptoms, and the expectations of the owners. Although canine AD cannot be cured, much progress has been done in recent years and more treatment options are available to improve the quality of life of affected patients.

## 9. Feline AD

Considerable debate still exists about the actual existence of AD in cats, as it does in other species. Feline allergic skin diseases tend to manifest in peculiar ways compared to other species. While dogs develop AD, which has strikingly similar characteristics to their human counterparts, the same does not seem to apply to cats with allergic skin diseases. Allergic skin disease triggered by environmental allergens and IgE mediation does exist in cats [47], but the clinical characteristics are peculiar for this species and no exact match exists with AD of other species. Additionally, very little is known about the skin barrier and impairment in cats; thus, AD in cats is still poorly investigated compared to what is known in other species.

Allergic skin diseases have been somewhat artificially divided based on the trigger factor, into flea-induced, food-induced, and environmentally-induced. The non-flea non-food cases have been, by default, frequently referred to as “atopic dermatitis”, although the clinical characteristics in cats are different from what is described under this name in other species. While some features apply (e.g., chronic, recurrent, pruritic dermatitis with familial predilection and, in most cases, associated with allergen-specific IgE), the clinical manifestations are quite peculiar (e.g., indolent ulcer, miliary dermatitis, eosinophilic granuloma complex). In humans and dogs, some areas are typically affected, such as flexural surfaces, and skin barrier dysfunction is believed to play an important role. In cats, studies on the skin barrier are minimal and, at this point in time, skin barrier defects have not been described in this species. The distribution of lesions also does not appear to mimic what is known as AD in other species, although the face is frequently affected, as seen in people and dogs (Figure 2). Frequently, the name AD is commonly used to describe allergic skin disease in which an environmental component is thought to play a role.

Clinically speaking, however, no characteristic pattern has been detected in those cases compared to the ones caused by flea and food. Thus, from the clinical standpoint, it is important to control insect exposure and rule out food-induced dermatitis before coming to the conclusion of “atopic dermatitis” caused by environmental allergens.

In a recent retrospective study, the prevalence of feline non-food, non-flea hypersensitivity was estimated to be 12%. The face and ventral abdomen were the most commonly affected and, in most cases (68%), allergen-specific IgE were detected on intradermal skin tests, suggesting that most cases, although not all, had an allergic component. Contrary to dogs and humans, Staphylococcal infections are not as common and reported in less than 50% of the patients. The diagnosis of “atopic dermatitis” in cats is a diagnosis of exclusion when no response is seen with insect control and dietary trials and, under this name, various clinical presentations can be included. It is unclear why some individuals develop granulomas or plaques, while others develop miliary dermatitis or self-induced alopecia. Histopathology does not help establish the triggering cause or identify the most appropriate therapy. The role of IgE has been supported in feline allergic skin disease as the injection of anti-IgE in healthy cats reproduced gross and cellular responses similar to what was found in clinical samples from affected patients.

When the microbiota of normal and allergic cats was compared, it was found that allergic feline skin had significantly greater amounts of Agaricomycetes and Sordariomycetes, and significantly fewer Epicoccum compared to healthy feline skin [48]. The skin of healthy cats appears to have a more diverse fungal microbiota compared to previous studies, and a fungal dysbiosis is noted in the skin of allergic cats.

Many of the same treatment strategies used in dogs and humans (e.g., cyclosporine, antihistamines, and immunotherapy) are used in cats to control clinical signs. Some cats also develop respiratory signs, like in atopic humans that develop asthma. The reasons for which some individuals only develop cutaneous disease with atopy, while others also have respiratory signs, are currently unknown. It is concluded that much work still needs to be done to understand the spectrum of diseases called atopic dermatitis in cats.

Identification of new therapies in cats with allergic skin disease is lagging behind compared to what is available in dogs. Currently available treatment options involve the use of anti-inflammatory and immunomodulatory agents, such as glucocorticoids and cyclosporine, as well as the control of triggering factors, such as insects and food allergies. All cats with non-seasonal symptoms should undergo a strict dietary trial with a hypoallergenic diet to elucidate the role of the diet in the clinical signs. Choices for a hypoallergenic diet range from new sources of proteins (e.g., rabbits, venison, and duck) to hydrolyzed diets. It is important to note that some patients may require more than one dietary trial, possibly due to different preservatives and hidden ingredients that may be different from one brand to another. Unfortunately, no laboratory testing is accurate to establish a diagnosis of food allergy.

For cases that do not respond to insect control and dietary trials, the clinician may attempt allergy testing, either by intradermal skin testing or serology testing. Intradermal skin testing is traditionally considered more specific, although it may be more technically challenging in cats than in other species. Factors that may suppress skin test reactivity include stress and the thinner dermis of felines, which make the injections more challenging in this species. If skin test cannot be accomplished serology may be considered and positive reactions should be correlated with seasonality of clinical signs. Allergy testing should not be used for diagnostic purposes, but only to select allergens to use for immunotherapy. Allergen-specific immunotherapy should be attempted in young individuals with a long season as a long-term strategy to decrease the need for rescue medications. Allergen-specific immunotherapy can be accomplished both by subcutaneous injections or the sublingual route. Efficacy of immunotherapy is typically not observed for the first six months of treatment and should be continued for a year before it can be fully assessed. For short-term control of clinical signs glucocorticoids are frequently used. For cats that do not tolerate glucocorticoids or have shown decreased response, oral cyclosporine may represent a suitable alternative [49]. The time to observe the response to therapy is typically longer with cyclosporine than with glucocorticoids. The most common adverse effect in cats receiving cyclosporine is vomiting, retching, and regurgitation (35%), followed by weight loss (20%) and diarrhea (15%). Thus, monitoring of body weight is recommended in cats on cyclosporine. Overall, adverse effects are observed in 60% of cats. Cyclosporine should not be used in cats with a history of malignant disorders, suspected malignancy, and cats infected with feline leukemia virus or feline immunodeficiency virus. The effect of cyclosporine on allergen-specific immunotherapy has not been studied in dogs and cats. No products aimed at skin barrier repair have been tested in cats as the role of the skin barrier in feline allergies is unknown. One published study reported the efficacy of oclacitinib in 5/12 allergic cats treated at the same dose used in dogs, although this drug is currently not approved for use in this species [50].

## 10. Equine AD

Horses are also affected by atopic disease and develop both cutaneous and respiratory signs. These diseases are IgE-mediated and are triggered by allergen exposure [51]. Cutaneous disease ranges from urticaria to AD and is frequently associated with insect hypersensitivity, which significantly contributes to the severity of the clinical signs. Some horses have both manifestations of atopic disease (skin and respiratory), while others have one or the other. Anecdotally, a progression from cutaneous to respiratory disease has been reported, but it is not clearly documented as it is in the atopic march in people. What determines the difference in the target organ or whether some individuals progress or not, is unclear at this time. Some allergic horses appear to also have a food allergy component. For example, some grasses that are high in protein content, like alfalfa, may be triggers for atopic disease both as food and as pollen.

As reported in humans and dogs, ultrastructural abnormalities in lipid lamellae and disorganization of the stratum corneum have been reported also in horses [52] and may be responsible for skin barrier impairment. Currently, no study has specifically addressed the permeability of equine atopic skin and compared it to the one of healthy controls or horses with other skin diseases. Recently, phospholipid abnormalities have been reported in the sera of allergic horses and appear to correlate with disease expression as they normalize when the disease is in remission [53].

Clinical signs of AD in horses include hives and/or pruritus that target the face (Figure 2), ears, antebrachial area, and the inguinal region. When patient have a concurrent *Culicoides* hypersensitivity, pruritus of the mane and tail, as well as of the legs and ears, further complicate the clinical presentation. Frequently, affected horses develop secondary staphylococcal infections that lead to increased pruritus and development of folliculitis and hair loss. It is important to note that, as in other species, allergy testing cannot be used for diagnostic purposes; allergic horses are more likely to have positive results on intradermal skin testing and have more severe reactions, although positive results may be seen in normal horses. Thus, intradermal skin testing cannot be used as a diagnostic test in atopic horses [54,55]. Variability in the performance of various serology tests has been documented in horses [56]. Thus, the choice of the company to use for serology testing may affect the composition of the immunotherapy.

Management of atopic horses requires the identification and correction of all factors playing a role in the pruritus, such as concurrent allergies and secondary infections [57]. The short-term approach involves the use of systemic and topical glucocorticoids. Orally, both prednisolone and dexamethasone can help decrease atopic pruritus. Prednisolone is a safer option for prolonged use, while dexamethasone is considered more likely to precipitate laminitis in predisposed individuals. Other oral therapies used to decrease inflammation and pruritus include antihistamines, like hydroxyzine or diphenhydramine and pentoxifylline. These therapies are rarely effective as monotherapy and typically require a combination to provide significant relief. Additionally, they appear to work better when initiated before the beginning of allergy season rather than in the midst of a flare. For long-term management and for horses with a long season, allergen-specific immunotherapy is recommended. Allergen-specific immunotherapy is safe and very effective in horses leading, in most cases, to discontinuation of other therapies and maintenance of remission solely with ASIT [58]. This approach is particularly helpful in patients that suffer from both cutaneous and respiratory disease.

## 11. Humans AD

In humans, AD (also known as eczema or atopic eczema) is a complex, chronic, inflammatory skin condition, affecting up to 10% of children and 4% of adults, with some geographic and ethnic differences [59,60]. Several studies have now confirmed the strong negative impact AD has on patients (and arguably on the families) quality of life. Pruritus and cutaneous infections are major drivers of the reduced quality-of-life associated with this disease. In the World Health Organization 2010 Global Burden of Disease survey, AD has ranked first among skin diseases [61].

Similarly to canine AD, hallmarks of the disease are epidermal barrier impairment and an abnormal immune response (Th2-predominant in acute lesions) to environmental allergens/antigens. About 80% of AD patients (so-called extrinsic) have high serum IgE levels, develop other allergic disorders at some point in their life, and can have positive prick tests to foods or aeroallergens. The intrinsic (non-allergic) form is clinically indistinguishable from the extrinsic form, but patients have normal levels of IgE, no identifiable allergic triggers, and negative prick tests.

In humans, as discussed above for other species, AD is a clinical diagnosis based on patient medical history, clinical findings, and exclusion of other cutaneous disorders (e.g., cutaneous T-cell lymphoma in adults/elderly) [62]. In the acute flare the primary lesions are intensely pruritic, erythematous macules or papules with a typical age-dependent distribution. In young children the face, scalp, and extensor surfaces of the arms and legs are more typically involved while, in older children, lesions more often are within flexural areas of the legs and arms. In the elderly, flexural areas might be spared. In addition to acute lesions, patients often present with excoriated papules with crust and serum exudates (secondary lesions to the intense itch) as well as lichenification of skin (chronic lesion).

In contrast to other species, in humans AD is often the first clinical manifestation of allergic disorders (so-called Atopic March) and it is followed by food allergy, rhinitis, and asthma (Table 1). Recent studies suggest that more than 50% of young children with severe AD will develop asthma and approximately 75% will develop allergic rhinitis [63].

In addition to the classic definition of extrinsic versus intrinsic AD, other stratification strategies have been proposed to better capture the complex pathophysiology and the wide spectrum of clinical phenotypes [64]. For example, age-based stratification, as proposed by Bieber et al., might allow to predict the possible natural history of the disease based on the disease onset. Based on this model, about 60–80% of all forms of AD start early in life (<2 years), and while a subgroup of children will likely “outgrow” the condition, about 40% will go on to have persistent disease in life. Children that present with early onset (2–6 years of age) are another subgroup of AD patients that most likely will continue to have AD as they grow. Adult onset has been shown for about 20% of the overall AD population. Interestingly, in this group are mainly female patients with a mild clinical phenotype and a very limited spectrum of sensitization, usually accompanied by a normal total IgE level. Very late onset (>60 years) AD is the subgroup that has been under-recognized for long time. This group included subjects that had AD in the past but had a longer period of remission and those who start d’emblee very late in life. These elderly AD patients often present with a severe (and hard to control) form of the disease, with diffuse eczematous up to erythrodermic lesions, and high total IgE levels.

All together these epidemiologic observations stimulate interesting research questions: Why do some patient outgrow the disease? Are there biomarkers or genetic traits that might help identify the different phenotype early in life? Can any early intervention change the natural history of the allergic disorders? One intriguing hypothesis is that sensitization through the skin early in life is critical for the pathogenesis of allergic disorders. Based on this hypothesis, it is exciting to speculate that early treatment of AD or early skin barrier repair could prevent development of the other allergic conditions later in life.

## 12. Skin Barrier Defects in AD and Allergic Disorders

As discussed for canine AD, skin barrier defects play an important role in AD pathogenesis in humans and possibly in other allergic disorder [65]. An important aspect of the AD barrier defects in humans (and dogs) is that it extends to non-involved or “normal appearing” skin and not only to eczematous areas. Clinical clues of barrier defects in AD include xerosis (skin appears dry), a reduced irritancy threshold, a key role of emollients in AD management, as well as increased TEWL and pH, and reduced skin hydration [66]. Additional, rare human genodermatosis provides interesting evidence of the association between epidermal barrier defects and allergies [67]. Netherton syndrome (NS), a rare autosomal recessive disorder, is due to a mutation in the epidermal gene *SPINK5* encoding the proteases inhibitor protein LEKTI. Netherton syndrome (NS) patients present with elevated IgE and have a high incidence of AD, asthma, allergic rhinitis, and food allergies [67]. More recently, mutations in the *desmoglein 1* gene have been associated with severe dermatitis, multiple allergies, and metabolic wasting (SAM) syndrome [68].

Findings from human clinical trials have recently provided important evidence of the association between barrier function and the development of allergic diseases. Two randomized, controlled, parallel-group studies conducted in the USA, Europe, and Japan have shown that the application of no medicated moisturizing early in life in children with a high risk to develop AD (e.g., strong family history) had a significant protective effect on the cumulative incidence of AD in the treatment group as compared to placebo.

A birth cohort study performed in the UK included 1903 infants recruited from 2009–2011. TEWL was measured at day 2, two and six months, and children were screened for food allergies at two years [69]. Remarkably, the authors found that neonatal skin barrier dysfunction predicts food allergy; day 2 upper-quartile TEWL was a significant predictor factor for food allergy at two years of age (OR 4.1; 95% CI, 1.5–4.8). This data supports the hypothesis of transcutaneous allergen sensitization for food allergy even in infants who do not have AD.

Similarly to canine AD, the SC is dysfunctional in AD subjects due to alterations in lipid composition, altered expression and function of epithelial-derived proteases, reduced expression of structural proteins (e.g., loricrin, filaggrin), and/or simply due to the mechanical actions of scratching. Tight junction (TJ) integrity and composition are also impaired in patients with AD. One of the most profound discoveries in the past decade in the field of human AD is related to filaggrin (FLG) loss of function mutations as a major predisposing factor for AD, and a possible role in other allergic disorders [70]. Several FLG mutations have now been discovered in several populations worldwide. Carriers of FLG mutation have greater risk to develop AD, peanut allergy, and asthma (but only in patients with concomitant AD) [71]. A recent study also found that FLG null mutations are associated with earlier AD onset in a dose-dependent manner (e.g., patients with two mutations vs. one mutation) [71]. However, FLG mutations have shown in up to 10% of non-atopic subjects in an Irish cohort, as well as in patients with ichthyosis vulgaris without AD, thus suggesting that other factors, in addition to FLG, must play a role in the pathogenesis of this complex disease [71].

## 13. Immunology in Humans AD

Studies in human samples (skin and serum) have contributed to characterizing the immune profile in AD skin. Following the first evidence of an increased expression of IL4 and IL13 in AD skin in 1994 [72], it is now well established that, in AD, there is a predominant Th2 immune response. However, other T helper cell subtypes have been identified in AD skin. Very intriguingly, the relative difference in the abundance of each Th-subgroup has been described not only in acute (e.g., Th22/Th17) vs. chronic (e.g., Th22/Th1) lesions, but also in adults vs. pediatric (e.g., > innate and IL17-related inflammation) patients, and based on ethnicity (e.g., Th17/22 > in Asian populations) [73]. Although, the pathogenic role of each of these additional Th-subgroups has to be fully clarified, it is possible to speculate that a better understanding of the different player could lead to better treatment plan. The central role of Th2 inflammation has been known for a long time in human allergic diseases, but it has been further confirmed by the positive effect of treatment with biologics targeting the Th2 axis (e.g., Dupilumab).

As conferred for canine AD, there is a strong crosstalk between epidermal barrier and atopic inflammation. The overall hypothesis is that epidermal barrier impairment allows the allergen/antigen to reach the resident antigen-presenting cells, thus eliciting an inflammatory response that, in the case of atopic individuals, is predominantly Th2. Importantly, epithelial barrier disruption in humans, as in dogs and murine models, is associated with the production of several pro-Th2 mediators (e.g., TSLP, IL-33, IL-25), which play a pivotal role in the initiation of allergic inflammation. Interestingly, genetic variants in the *TSLP* gene have been linked to AD persistence and eczema herpeticum [74].

On the other hand, recent studies using human keratinocytes have identified a number of T-cell derived cytokines found in AD skin (e.g., IL-4, IL-13, TNF-α, IL-25, IL-22, or IL-17A) can inhibit the expression of key epidermal barrier proteins (e.g., filaggrin, loricrin, S100A11, and involucrin). This creates a vicious cycle between the barrier and inflammation that most likely supports the chronicity of the disease. A better understanding of the complex interactions between relevant T helper cytokines found in AD skin and how they affect epidermal barrier function will be critical for a better understanding of AD pathogenesis and will hopefully result in new targeted therapeutics.

## 14. Human AD and Infections

Similarly to canine AD, Staphylococcal infection and colonization play a critical role in the diseases pathogenesis. As discussed earlies for dogs, a lack of microbiome diversity has been found in AD subjects as it compared to non-atopic controls [75].

One characteristic of atopic humans, which is not observed in atopic animals, is the propensity for viral infections. Eczema herpeticum (EH) is a rare, but potentially life threatening, complication of human AD [76]. EH is a widespread viral infection in patients with eczema and, potentially, other forms of dermatitis (e.g., pemphigus vulgaris or psoriasis). More frequently EH is due to a primary HSV1-2 infection in young AD patients.

## 15. Therapeutic Approach

As discussed for the canine AD, there are two-fold goals for the treatment: control of acute flares and long-term management. Sadly, despite its high prevalence, the effects on quality-of-life, and economic burden, there are few effective treatments for AD.

Moisturizers, gentle skin care, and trigger (irritants, allergens, heat, stress, etc.) avoidance are the cornerstone of AD treatment in humans, indicated as primary therapy in mild cases and necessary support for more severe cases. Ointments tend to have the greatest moisturizing effect, followed by creams, and then lotions. Moisturizing should be fragrance free. Patient (and family) education on proper skin care is essential for an optimal management of the disease. Topical steroid and/or calcineurin inhibitor (tacrolimus and pimecrolimus) are widely used to control acute flares and as proactive treatment (2–3 times a week) to prevent relapses in patients with mild–moderate disease [77]. Topical calcineurin inhibitors were approved by FDA in 2005 for AD patient over two years of age. More recently, a topical Phosphodiesterase (PDE) 4 inhibitor has been approved by FDA for children (over two years of age) and adults with AD. For more recalcitrant cases wet wrap therapy is often used in conjunction with the topical anti-inflammatory products [78].

As mentioned initially, pruritus and infection are important drivers of acute flares and poor quality of life in AD patients. The use of anti-histamine is somehow still controversial in AD. Often, sedative anti-histamines are used as sleeping aid to provide patients some relief at night time. However, there is no strong evidence they help to control the itch. This is in line with several studies clearly showing that, in addition to histamine, there are several other mediators/receptors involved in the pathogenesis of the atopic itch [79]. Currently, there are several drugs in human clinical trials that are promising to help control the atopic itch. Interestingly, some of these drugs have also been tested, or are already available, for canine patients. For example, the results from a Phase II trial with Nemolizumab, a humanized antibody against interleukin-31 receptor, have been recently published and showed a significantly improved pruritus in patients with moderate–severe AD with an overall good safety profile [80]. Treatment of acute infection with topical or systemic antibiotic is recommended, as well as anti-virals for the treatment of EH. Recent studies also recommended the use of anti-fungals in patients with evidence of *Malassezia* species infection (e.g., positive KOH, specific IgE). A bit more controversial are anti-microbial proactive regimes in patients with recurrent infections, mostly due to the concerns for antibiotic resistance. A recent study by Huang et al. [81], showed intermittent intranasal mupirocin application in conjunction with diluted bleach baths (2–3 times per week) reduced the clinical disease severity in AD patients with evidence of bacterial infection.

The use of ASIT in human medicine, at this point in time, is primarily used for allergic asthma, rather than for AD, although some preliminary case series also show promise for the treatment of AD [82,83].

When topical and lifestyle modifications do not provide the desired clinical improvement, systemic therapy is recommended. Phototherapy (more frequently narrow-band UVB), systemic corticosteroids, or other broad immunosuppressant (e.g., cyclosporine, MTX, Cell Cept), can be used in AD patients based on specific patient age, medical history, cost, and availability [84]. Importantly, at least in the USA, these systemic immunosuppressant medications are used off-label (i.e., no FDA indication for AD), mainly for the lack of randomized clinical trials. In several European countries only short courses of Cyclosporine are approved by the specific regulatory agency.

In March 2017, the FDA approved Dupilumab the first (ever) biologic for patients with moderate-to-severe AD who cannot be controlled with topical medications. This is a fully human monoclonal antibody targeting the IL-4 receptor alpha subunit (IL-4Rα), thus blocking the intracellular signaling of both IL-4 and IL-13 (Th2 cytokines). Results from Phase III studies showed Dupilumab (vs. placebo) induced significant improvement of clinical scores (Eczema Area and Severity Index [EASI] and Investigator Global Assessment [IGA]), as well improvement in pruritus with an overall good safety profile. After an initial loading dose (300 mg × 2) the drug is given every two weeks. Although Dupilumab has been approved, for now, in patients 18 years of age and older, trials are ongoing in pediatric populations. This is extremely exciting as it will not only provide the younger patients with an effective treatment with potential less of the side effects of the more traditional medications, but these trials might help shed some light on the role of the Th2 pathway in the natural history of the allergic disease progression.

Numerous biologics, as well as small-molecule drugs, are currently under investigation for AD in people [85]. Among those, it is worth briefly mentioning Janus Kinase (JAK) inhibitors for the comparative aspect. Unlike in dogs, where the efficacy of Oclaticinib has been already demonstrated in the clinic, in humans JAK inhibitors are currently been tested in clinical trials. In particular, topical formulations are particularly interesting in AD patients as they could provide clinical improvement with limited toxicity. Data from a Phase IIa trial, showed that topical Tofacitinib (JAK 1 and 3 inhibitor) induced significant improvement of the clinical scores by week 1 and itch by day 2 post treatment vs. vehicle [86].

This is an exciting time for translation research in AD, with numerous trials underway, as well as basic science investigations.

## 16. Conclusions 

AD is a multifactorial clinical syndrome that can be diagnosed in multiple species. The burden of this disease is significant in both animals and humans in terms of quality of life and propensity toward infections, which leads to frequent antibiotic use and increased risk for development of resistance. Similarities between animals and humans involve the propensity for epicutaneous allergic sensitizations, the aggravating role of secondary bacterial infections and the need to control pruritus by using a multimodal approach. Glucocorticoids and other anti-inflammatory therapies are used to provide relief. There are still unmet needs in terms of identification of therapies that have broad coverage, yet minimal adverse effects. Leveraging the differences between species (e.g., the lack of progression to asthma in atopic dogs) may help us understand the factors that play a role in the progression of the disease. Some treatments that are used in veterinary medicine are not available in human medicine, and vice versa. It is helpful for us to learn from the experience in other species to broaden our approach and our understanding of this complex syndrome.

## Figures and Tables

**Table 1:** Characteristics of atopic dermatitis across species.

Characteristics	Dogs	Cats	Horses	Humans
Age of onset	Young adults (1–3 years of age)	Young adults (1–3 years of age)	Young adults (1–2 years of age)	60% Early onset (< 2 years of age) 20% adult onset
Progression of atopic march	Extremely rare	Rare	Not uncommon	Common
Course of disease	Chronic progressive	Chronic progressive	Chronic progressive	Chronic and relapsing progression.
Presence of IgE	80% of cases	60% of cases	95% of cases	70–80% of cases
Skin barrier defect	Documented decreased ceramides, discontinuous lipid lamellae, abnormal filaggrin expression, changes in expression of tight junction proteins, and increased transepidermal water loss. No documented mutation linked to AD.	None documented	Documented alteration of lipid lamellae and ultrastructure of the stratum corneum. No documented mutation	Documented decreased ceramides, discontinuous lipid lamellae, abnormal expression of epidermal differentiation complex components (including reduced expression of filaggrin and loricrin), impaired tight junction function and reduced expression of key components (claudin-1), increased water loss (non-lesional and lesional)
Prevalence	20–30%	10–20%	20–40%	10% of children and 4% of adults
Association with other diseases	Food allergy, Flea allergy	Food allergy, Flea allergy	Culicoides hypersensitivity	Food allergy, asthma, allergic rhinitis
Staphylococcal infection/colonization	Extremely common	Uncommon	Common	Extremely common
Most common therapies	Oral and topical glucocorticoids, JAK inhibitor, cyclosporine, antihistamines, Allergen specific immunotherapy, IL31 monoclonal antibody	Oral and topical glucocorticoids, antihistamines, cyclosporine, allergen specific immunotherapy	Oral and topical glucocorticoids, antihistamines	Moisturizing, gentle skin care and avoidance of triggers. Oral and topical glucocorticoids. Phototherapy (nbUVB). Immunomodulants (e.g., cyclosporine, methotrexate, mycophenolate mofetil). Dupilumab (fully human monoclonal antibody targeting the IL-4 receptor alpha subunit)
